# Growth Promotion of Delayed-Transplanted Strawberry Plug Seedlings by Single- and Combined-Strain Inoculation of *Bacillus* spp.

**DOI:** 10.3390/microorganisms14102135

**Published:** 2026-09-22

**Authors:** Feng Liu, Likai Zhang, Huifang Zhao, Jianli Xia, Sijian Guo, Chunzhen Cheng

**Affiliations:** 1 Shanxi Agricultural University, Taiyuan 030031, China; 2 Shanxi Key Laboratory of Germplasm Improvement and Utilization in Pomology, Taiyuan 030031, China

**Keywords:** *Bacillus*, delayed transplanting, growth and development, metabolome, strawberry

## Abstract

Delayed transplanting of plug seedlings frequently causes root coiling and growth retardation in strawberry (*Fragaria* × *ananassa*), compromising transplant quality and commercial nursery performance. While plant growth-promoting bacteria (PGPB) are widely used to enhance crop growth and resilience, their effects on delayed-transplanted strawberry remain poorly understood. This study investigated the individual and combined effects of two *Bacillus* strains (KP3P9 and K13C) on the growth and root architecture of 12-week-old ‘Benihoppe’ strawberry plug seedlings, which had been delayed-transplanted for approximately 5–8 weeks. Moreover, root metabolomic profiles were analyzed to uncover metabolic reprogramming associated with *Bacillus* inoculation. Results showed that all *Bacillus* treatments promoted plant growth, enhanced fresh weight, and improved root development of delayed-transplanted strawberry. Metabolomic analysis revealed that all *Bacillus* treatments increased steroid levels but decreased flavonoids, ketones/aldehydes/esters, lignans, nucleotides, phenylpropanoids, and sugars in roots of delayed-transplanted strawberry plants. Furthermore, distinct metabolic signatures were found among treatments: the single KP3P9/K13C treatment elevated polyphenols and terpenoids; K13C uniquely increased alcohols, alkaloids, and coumarins, while their co-application (KK) specifically enhanced amino acid accumulation. KEGG enrichment analysis further revealed varied metabolic reprogramming: KP3P9 significantly enriched pantothenate and CoA biosynthesis, cornified envelope formation, and aminoacyl-tRNA biosynthesis; K13C enriched tyrosine metabolism and alpha-linolenic acid (ALA) metabolism, whereas KK specifically enriched flavonoid metabolism. This study reveals the promoting effects of single and combined KP3P9 and K13C applications and provides novel insights into the metabolic basis of *Bacillus*-mediated growth enhancement on delayed-transplanted strawberry.

## 1. Introduction

Plant growth-promoting microorganisms (PGPMs) comprise diverse bacteria and fungi that colonize plant roots or the rhizosphere, enhancing host growth and productivity under both favorable and stressful conditions [1,2]. Among them, the genus *Bacillus* stands out as a prominent group of plant growth-promoting rhizobacteria [3]. *Bacillus* species are valued for their ability to form resistant endospores [4], ensuring long-term formulation stability, as well as their metabolic versatility and strong rhizosphere competence [5,6]. They can promote plant growth through multiple mechanisms, including biological nitrogen fixation, phosphate solubilization, and siderophore-mediated iron acquisition [7]; phytohormone biosynthesis that stimulates cell division [8]; root architectural remodeling and rhizosphere microbiome optimization [9,10]; enhancement of antioxidant defenses [11]; and improved nutrient-foraging capacity [12]. Recently, accumulated evidence revealed that the *Bacillus*-plant interactions were also involved in the reprogramming of plant metabolomes [13]. *B. megaterium* BT22 promoted Arabidopsis growth by regulating plant hormone homeostasis and inducing metabolome reprogramming. Its application greatly induced the contents of nine flavonoids and seven phenylpropanoid metabolites in Arabidopsis [14]. The co-application of *B. subtilis* and water-retaining agents on wheat greatly influenced the flag leaf metabolome, with metabolites involved in ABC transporters and amino acid biosynthesis significantly enriched [15]. Cucumber plants inoculated with *B. subtilis* MBI600 exhibited a higher abundance of the sugars fructose, glucose, and mannose, as well as the sugar alcohol myo-inositol in leaves at 24 h post inoculation (hpi), while at 48 hpi, the amounts of sugars decreased, but the majority of amino acids increased [16]. *B. velezensis* ES2-4 inoculation led to the upregulation of antifungal metabolites (e.g., oxalic acid and eicosane) in root exudates [17]. These results indicated that the growth-promoting and stress-enhancing abilities of *Bacillus* species were achieved through metabolome reprogramming to some extent.

Strawberry (*Fragaria* × *ananassa*) is a perennial dicotyledonous herbaceous plant of the genus *Fragaria* in the Rosaceae family [18]. In recent years, with the continuous expansion of the cultivation area and the increase in market recognition, strawberry has become one of the most popular and economically important fruit crops worldwide [18,19]. The widespread adoption of plug-tray substrate propagation has enabled large-scale nursery production and facilitated the use of tabletop cultivation systems [18]. This method offers numerous advantages, including high survival rates, absence of transplant shock recovery periods, and earlier fruit maturity. Compared with traditional bare-root seedlings, plug transplants offer higher survival rates and accelerated fruit maturity [20]. However, transplant timing is critical for the growth and development of strawberry plug seedlings. The optimal window occurs approximately 4–7 weeks after plugging, when roots have fully colonized the plug cell volume [21]. Delayed transplanting diminishes or negates the advantages of plug seedlings [22], leading to root penetration through the bottom drainage holes and subsequent root coiling within the tray, a phenomenon known as ‘root binding’ or ‘plug-bound root’. Overgrown root systems make extraction from plug trays difficult during field establishment, increasing the risk of substrate loss and mechanical root injury, thereby compromising transplant quality. This situation engenders a cascade of production problems, including root damage, reduced growth vigor [23], and delayed floral bud differentiation [24,25]. Moreover, delayed transplanting reduces fruit marketability and negatively impacts economic returns.

Numerous studies have demonstrated the benefits of *Bacillus*-based inoculants in strawberry cultivation, including improved fruit yield, photosynthetic efficiency, and postharvest quality [26,27,28]. *Bacillus*-based PGPM treatments enhance both productivity and postharvest fruit quality in the ‘Clery’ cultivar [29]. Co-inoculation of *B. subtilis* and the arbuscular mycorrhizal fungus *Rhizophagus intraradices* boosts vegetative growth and improves fruit yield and the quality of ‘San Andreas’ strawberry [30]. *B. velezensis* TCS001 not only stimulates plant growth but also provides control efficacy against anthracnose disease caused by *Colletotrichum gloeosporioides* [31]. Likewise, both *B. velezensis* and *B. subtilis* effectively preserve root integrity, support robust plant development, and enhance strawberry resistance to *Neopestalotiopsis clavispora*-caused root rot disease [32]. Although accumulated studies reveal the considerable potential of *Bacillus* species for enhancing strawberry cultivation, their efficacy in promoting the growth of delayed-transplanted strawberry seedlings and their influences on the root metabolome remain to be investigated.

In our previous study, two *Bacillus* strains (KP3P9 and K13C) isolated from apple rhizosphere soil were identified as having the ability to promote the growth and enhance stalk rot resistance of maize seedlings [33]. Both strains exhibited inorganic phosphate-solubilizing capacity, along with protease and siderophore production; K13C additionally displayed organic phosphate-solubilizing and potassium-releasing activities [33]. In the present study, we applied these two strains to 12-week-old ‘Benihoppe’ strawberry plug seedlings (delayed-transplanted for 5–8 weeks). The effects of individual inoculations with KP3P9 (K9 group) and K13C (KC group), as well as combined inoculation with KP3P9+K13C (KK group), on the growth and development of these delayed-transplanted plants were investigated, with untreated delayed-transplanted seedlings as the control (CK group). To further explore the influences of K9, KC, and KK treatments on the root metabolomes of delayed-transplanted strawberry plants, non-targeted metabolomic analysis of roots across CK, K9, KC, and KK groups was conducted. Our study will reveal not only the effects of *Bacillus* treatments on the growth of delayed-transplanted strawberry plants but also provide insights into the root metabolic changes associated with these treatments.

## 2. Materials and Methods

### 2.1. Plant and Microbe Materials

Uniform and healthy runner plantlets were harvested from stock ‘Benihoppe’ strawberry (*Fragaria* × *ananassa* Duch.) plants and pruned to retain only two leaves. After soaking in Amistar (azoxystrobin) solution (Syngenta Group Co., Ltd., Shanghai, China; diluted 1000-fold) for 5 min and then rinsing in water for 30 min, runner plantlets were stored at 4 °C in the dark for 3 days to induce rooting prior to plug-tray planting. Then, they were cultivated in cultivation substrates (charcoal: vermiculite: perlite = 5: 3: 2, *v*/*v*/*v*) in 24-cell plug trays (cells were of upper diameter of 6 cm, bottom diameter of 2.4 cm, and height of 6 cm) in a solar greenhouse located at a strawberry family farm (35°42′7.84″ N, 111°38′49.90″ E) in Yicheng County, Linfen City, Shanxi Province, China. Plug seedlings were irrigated with water once a day, and the Yamazaki strawberry-formula nutrient solution (diluted approximately 4-fold with water, electrical conductivity (EC) controlled at 0.4–0.6 mS/cm, and pH at 6.0–6.5) [34] was supplied once a week. No pesticides, fungicides, or other fertilizers were applied during plug production. During the cultivation of these plug seedlings, the temperature in the solar greenhouse ranged from 10 °C (the lowest temperature at night) to 25 °C (the highest temperature during the day), with relative humidity of 60–70%. The optimal transplanting time for plug seedlings was approximately 4–7 weeks after plugging [21]. Therefore, the seedlings used in the present study (12-week-old) had been delayed-transplanted for approximately 5–8 weeks. The two plant growth-promoting bacteria, *Bacillus siamensis* KP3P9 (K9) and *B. subtilis* K13C (KC), were previously isolated [33] and maintained at −80 °C in a 30% glycerin solution in our laboratory. Before use, the bacterial solution was streaked onto sterilized Luria–Bertani (LB) medium and incubated at 37 °C for one day.

### 2.2. Bacterial Suspension Preparation and Inoculation

Single colonies of KP3P9 and K13C were inoculated into sterilized LB liquid medium and incubated at 37 °C with shaking at 180 rpm for 24 h. The bacterial cultures were adjusted to an optical density (OD_600_) of 1.0 (approximately 5.0 × 10^8^ colony-forming units (CFU)/mL for both strains) with sterilized LB medium and then diluted threefold with sterile distilled water to obtain the inoculation suspensions.

Unique and healthy 12-week-old strawberry plug seedlings were selected and randomly divided into four groups. For each group, approximately 100 seedlings were used. Irrigation was withheld for two days prior to inoculation. Then, seedlings were inoculated with either KP3P9 suspension (K9 group), K13C suspension (KC group), a 1:1 (*v*/*v*) mixture of KP3P9 and K13C (KK group), or sterile threefold-diluted LB medium (CK group). For each seedling, 25 mL of bacterial inoculation suspension was inoculated into substrates around strawberry roots. Bacterial inoculation was repeated after 24 h, i.e., each plant was inoculated twice. Subsequently, the plug seedlings from each group (108, 92, 90, and 98 for the CK, K9, KC, and KK groups, respectively) were transplanted into cultivation substrates (charcoal: vermiculite: perlite = 5: 3: 2, *v*/*v*/*v*) in cultivation troughs (50 cm length × 26.5 cm width × 17.5 cm height). Six seedlings from the same group were planted in each cultivation trough. Seedlings in troughs containing fewer than six individuals were used only for survival rate assessment. All seedlings were cultivated in a temperature-controlled solar greenhouse (15–20 °C at night, 25–30 °C during the day, 60–70% relative humidity) equipped with integrated water and fertilizer management systems. When the temperature exceeds 30 °C, the ventilation of the greenhouse will be activated automatically to decrease the temperature. The light intensity in the solar greenhouse rose gradually from 6:00 (10~400 lux) to 12:00 (40,000~75,000 lux), peaked at 60,000~84,000 lux around 13:00, and then showed an overall declining trend, dropping to 0 lux after 19:00. Seedlings were irrigated with water once a day, and the Yamazaki strawberry-formula nutrient solution (diluted 2- to 3-fold with water, EC controlled at 0.6–0.8 mS/cm, and pH at 6.0–6.5) [34] was supplied once a week.

### 2.3. Measurements of Growth-Related Parameters

Three months after transplantation, the survival rate of strawberry seedlings in each group was calculated as follows: Survival rate (%) = (Number of surviving seedlings/Total number of seedlings per group) × 100. Plant height, crown size (the diameter of the crown), petiole length, and leaf number were then measured for 24 plants per group, with two plants randomly sampled from each of 12 selected independent cultivation troughs, using a ruler. Above-ground part fresh weight, root fresh weight, and plant fresh weight were determined using an electronic balance (model HAT-A+100; Huazhi (Fujian) Electronic Technology Co., Ltd., Putian, China), with six replications for each group.

### 2.4. Root Scanning

Six strawberry plants were randomly sampled from six independent cultivation troughs, gently washed with sterile water to remove adhering substrate, and then subjected to root system scanning [35]. Root architecture parameters, including total root length, average root diameter, root volume, root surface area, root projected area, and root tip number, were analyzed using a ScanMaker i800 Plus GXY-A root phenotyping system (Hangzhou Guangxiong Technology Co., Ltd., Hangzhou, China).

### 2.5. Co-Culture Assays of KP3P9 and K13C

To study the antagonism between KP3P9 and K13C, a cross-streaking assay was performed as described by Hossain (2024) [36]. Briefly, one strain (KP3P9 or K13C) was streaked along the center of LB agar plates using a sterile inoculating loop. The plates were incubated inverted at 37 °C for 12 h, after which the other strain was streaked perpendicularly across the first streak. Following further incubation at 37 °C for 16 h, the interaction phenotypes between KP3P9 and K13C were observed.

### 2.6. Metabolite Extraction

Fifteen strawberry plants from each group (three randomly sampled plants from each of five selected independent cultivation troughs) were selected for root harvesting. Roots were washed under running water to remove the attached substrate, rinsed three times with sterile water (1 min each), and blotted dry with sterile filter paper. The roots of five plants were pooled to form one biological replicate, with three replicates per group. Root samples were ground into a fine powder in liquid nitrogen and subjected to metabolite extraction [37]. Fifty milligrams of powder was extracted with 1 mL of extraction solvent (methanol/acetonitrile/water, 2:2:1, *v*/*v*/*v*) containing internal standards (20 mg/L), followed by vortexing for 30 s. Steel beads were added, and the samples were homogenized at 45 Hz for 10 min using a bead mill, followed by sonication for 10 min in an ice-water bath. The samples were then incubated at −20 °C for 1 h and centrifuged at 12,000 rpm for 15 min at 4 °C. The supernatant (500 μL) was transferred to a 1.5 mL microcentrifuge tube and dried in a vacuum concentrator. The dried metabolites were reconstituted in 160 μL of reconstitution solvent (acetonitrile/water, 1:1, *v*/*v*), vortexed for 30 s, and sonicated for 10 min in an ice-water bath. After centrifugation at 12,000 rpm for 15 min at 4 °C, 120 μL of the supernatant was transferred to a 2 mL autosampler vial for LC-MS analysis.

### 2.7. Non-Targeted Metabolomic Analysis

Non-targeted metabolomic analyses were performed on 12 root samples (three replicates per group) using an ultra-performance liquid chromatography coupled with quadrupole time-of-flight mass spectrometry (UPLC-QTOF-MS) platform [37]. The instrument consisted of a Waters Acquity I-Class PLUS UPLC system coupled with a Waters Xevo G2-XS QTOF high-resolution mass spectrometer (Waters Corporation, Milford, MA, USA). Raw data were acquired using MassLynx V4.2 and imported into Progenesis QI software (Version 2.4, Nonlinear Dynamics, Newcastle-upon-Tyne, UK) for automated alignment, deconvolution, and peak picking. Metabolites were annotated by searching against the Waters METLIN MS/MS database and public spectral libraries containing in silico MS/MS data. The identification algorithm employed a composite scoring system (maximum 60 points) based on three equally weighted criteria (20 points each): precursor ion mass accuracy, isotopic distribution fidelity, and MS/MS fragment ion matching. Mass tolerance windows were set to ±100 ppm for precursor ions and ±50 ppm for fragment ions. Only compounds with a total identification score of ≥40 out of 60 were retained. Based on core metabolite scaffolds, biosynthetic origins and classic biochemical categories, identified metabolites were assigned to distinct Class I-level categories for global metabolome distribution profiling. Fine-grained Class II-level subcategories under Class I were further defined based on scaffold modifications, ring-system architectures, branched biosynthetic pathways, and functional group subtypes. This two-tier nested classification uses a top-down structure, and each metabolite maps to exactly one Class I category and its corresponding Class II subcategory. Principal component analysis (PCA) of identified metabolites in roots of different groups was performed using the procomp function in R (version 3.6.1), and heatmaps were generated using the pheatmap package (version 1.0.2).

### 2.8. Identification and Enrichment Analysis of Differentially Accumulated Metabolites (DAMs)

Differentially accumulated metabolites (DAMs) between each treatment group (K9/KC/KK) and the CK group were screened based on the following criteria: variable importance in projection (VIP, obtained from the multivariate OPLS-DA model) ≥ 1, *p* < 0.05, and |log_2_(fold change (FC))| ≥ 1. Venn analysis of DAMs among different comparisons was performed using the VennDiagram package (version 1.6) in R. DAMs were mapped to the KEGG COMPOUND database, and pathways with *p* < 0.05 (Fisher’s exact test) were considered significantly enriched.

### 2.9. Statistical Analysis

All the data obtained in this study were compiled using Microsoft Excel and are presented as means ± standard deviations (SD, *n* ≥ 3). Following assessment of normality (Shapiro–Wilk test) and homogeneity of variance (Levene’s test), one-way ANOVA with Duncan’s post hoc test was performed using SPSS 27.0 (IBM, Armonk, NY, USA) to determine significant differences among the four groups at the *p* < 0.05 level.

## 3. Results

### 3.1. Effects of Bacillus Inoculation on the Growth of Delayed-Transplanted Strawberry

Three months post-transplantation, the survival rates of strawberry seedlings from the four groups were recorded. The survival rates of the CK, K9, KC, and KK groups were 92.59% (100/108), 98.91% (91/92), 100% (90/90), and 98.98% (97/98), respectively. Phenotypic observation showed that, compared with the *Bacillus*-treated groups, strawberry plants from the non-treated control (CK) group exhibited weaker root systems and fewer leaves, especially new leaves (Figure 1). This finding indicated that the three *Bacillus* treatments not only promoted root development but also enhanced the growth of the above-ground parts of delayed-transplanted strawberry plug seedlings.

Growth-related parameters of strawberry plants from the four groups were measured (Figure 2A). Results revealed that the average plant height in the K9, KC, and KK groups was significantly higher (*p* < 0.05), being 1.30-, 1.40-, and 1.53-fold of the CK group, respectively. The average crown size in the KC and KK groups was also significantly greater (*p* < 0.05), reaching 1.30- and 1.33-fold of the CK group, respectively. The K9 group likewise exhibited a larger crown size than the CK group (1.23-fold), although this difference was not statistically significant (*p* > 0.05). The average petiole length in the K9, KC, and KK groups was all greater than that in the CK group, with the KC group showing a significant increase (1.46-fold of the CK group) (*p* < 0.05). The average leaf number in the K9 and KC groups was significantly higher than that in the CK group (*p* < 0.05) (1.67- and 1.62-fold, respectively), while the KK group showed a 1.55-fold increase compared to the CK group (*p* > 0.05).

### 3.2. Influences of Bacillus Inoculation on the Fresh Weight of Delayed-Transplanted Strawberry

Fresh weight measurement results revealed that the root fresh weight, above-ground part fresh weight, and plant fresh weight of the K9, KC, and KK groups were all significantly higher than those in the CK group (*p* < 0.05) (Figure 2B), accounting for approximately 4.17-, 4.09-, and 3.22-fold, 5.99-, 6.21-, and 5.11-fold, and 5.10-, 5.17-, and 4.18-fold of CK, respectively.

### 3.3. Influences of Bacillus Inoculation on the Root Architecture of Delayed-Transplanted Strawberry

Root scanning was further employed to investigate the effects of *Bacillus* treatments on the root architecture of delayed-transplanted strawberry plants (Figure 2C). Results showed that the average root diameter in the K9, KC, and KK groups was significantly greater than that in the CK group (*p* < 0.05), being approximately 1.26-, 1.36-, and 1.25-fold of the CK, respectively. The root volume of the KC group was significantly higher than that of the CK and KK groups (*p* < 0.05), accounting for approximately 2.14-fold of the CK and 1.91-fold of the KK. Root surface area and projected area in the K9 and KC groups were significantly increased compared with the CK group (*p* < 0.05), both being approximately 1.87- and 1.92-fold of the CK group, respectively. In contrast, the root tip number in the KK group was significantly lower than that in the CK group (*p* < 0.05). These results indicated that the influences of *Bacillus* inoculation on the root architecture-related parameters of delayed-transplanted strawberry plants varied among single strains (K9/KC) and their combination (KK).

### 3.4. Cross-Streaking Assays of the Antagonism Between KP3P9 and K13C

Since KK exhibited weaker promoting effects on all measured parameters (except plant height and crown size) compared with single-strain treatments (KC and K9), it was hypothesized that there might be antagonism between KP3P9 and K13C. Consistently, cross-streaking assays revealed an inhibition zone between them, indicating that there is antagonism between the two *Bacillus* strains (Supplemental Figure S1). This finding indicated that the relatively weaker promoting effects of KK on the growth of delayed-transplanted strawberry were associated with the antagonism between the two *Bacillus* spp., while its better promoting effects on plant height and crown might be caused by the altered rhizosphere colonization, metabolite interactions, or distinct host plant responses.

### 3.5. Root Metabolomic Analysis of Delayed-Transplanted Strawberry

Evidence revealed that the influence of *Bacillus* spp. on the growth of host plants is involved in the reprogramming of plant metabolomes [13,14]. In this study, to explore the influences of the three *Bacillus* treatments on the root metabolomes of delayed-transplanted strawberry, non-targeted metabolomic analysis was further conducted. In total, we detected 16,963 peaks from strawberry roots from the four groups, based on which 4443 metabolites were successfully annotated (Supplemental Table S1). Strong correlations were found among biological replicates within each group (*r* > 0.993) (Figure 3A), indicating excellent reproducibility and reliability of the metabolomic data. Inter-group correlation analysis revealed that the K9 and CK groups exhibited the highest metabolomic correlation coefficient, ranging from 0.917 to 0.922, followed by the KC and CK groups (*r* = 0.875–0.881), while the KK and CK groups showed the lowest correlation (*r* = 0.810–0.815) (Figure 3A). These results indicated that the KK treatment exerted the greatest impact on the root metabolome. Principal component analysis (PCA) further demonstrated that the K9 group was positioned closest to the CK group (Figure 3B), indicating that K9 had minimal effect on the strawberry root metabolome.

Non-targeted metabolomic data from the CK, K9, KC, and KK groups were subjected to metabolite classification at the Class I level, encompassing 17 metabolite categories, including lipids, ketones/aldehydes/esters, terpenoids, and others (Figure 4). Comparisons of the total peak areas of these Class I-level metabolites across groups revealed that all three treatments significantly decreased the contents of flavonoids, ketones/aldehydes/esters, lignans, nucleotides, phenylpropanoids, and sugars (*p* < 0.05), while significantly increasing steroid content in strawberry roots (*p* < 0.05) (Supplemental Table S2). The K9 and KC treatments significantly elevated polyphenols and terpenoids contents (*p* < 0.05), and the KC treatment significantly increased alcohol content (*p* < 0.05), whereas the other groups showed no significant difference in alcohol content compared with the CK group (*p* > 0.05). Additionally, the KC treatment significantly increased alkaloids and coumarins contents (*p* < 0.05), while the K9 and KK treatments significantly decreased both (*p* < 0.05). Notably, the KK treatment specifically increased amino acid content in strawberry roots (*p* < 0.05). Furthermore, none of the three treatments significantly affected the total contents of lipids, organic acids, quinones, or vitamins. These results indicated that the three *Bacillus* treatments exerted distinct growth-promoting effects on delayed-transplanted strawberry plants, which may be attributable to their differential modulation of root metabolomes.

### 3.6. Identification of Differentially Accumulated Metabolites Among the Four Groups

Using the criteria of VIP ≥ 1 and *p* < 0.05, DAMs were identified in the CK_vs_K9, CK_vs_KC, and CK_vs_KK comparisons (Figure 5A). A total of 705 DAMs were identified in the CK_vs_K9 comparison (Supplemental Table S3), comprising 465 down-regulated and 240 up-regulated metabolites (Figure 5B). The CK_vs_KC comparison contained 1149 DAMs (Supplemental Table S4), with 547 down-regulated and 602 up-regulated metabolites (Figure 5C). The CK_vs_KK comparison exhibited the largest number of DAMs, totaling 1821 (Supplemental Table S5), including 1,348 down-regulated and 473 up-regulated metabolites (Figure 5D). These results indicate that the KK treatment exerted the greatest impact on the strawberry root metabolome, followed by the KC treatment, while the K9 treatment had minimal effect.

Venn analysis of DAMs in the CK_vs_K9, CK_vs_KC, and CK_vs_KK comparisons (Figure 5E) revealed that the CK_vs_KK comparison contained the highest number of unique DAMs (918), followed by the CK_vs_KC comparison (344), and the CK_vs_K9 comparison (63) (Supplemental Table S6), indicating substantial differences among the three treatments in their effects on the root metabolome. A total of 436 DAMs were shared among all three comparisons, with 315 DAMs shared between the CK_vs_KC and CK_vs_KK comparisons, 152 between the CK_vs_K9 and CK_vs_KK comparisons, and 54 between the CK_vs_K9 and CK_vs_KC comparisons. Among the 136 commonly up-regulated metabolites (Figure 5E, Supplemental Table S6), lipids and ketones/aldehydes/esters accounted for the highest proportion. Of the 275 commonly down-regulated metabolites (Figure 5E, Supplemental Table S6), terpenoids and lipids were the predominant categories, followed by ketones/aldehydes/esters and sugars.

By further analyzing the top 20 up/down-regulated DAMs in the three comparisons, we found that these metabolites mainly belonged to lipid, ketones/aldehydes/esters, terpenoids, polyphenols, organic acids, nucleotides, coumarins, and sugars (Table 1), indicating that *Bacillus* treatments greatly influenced the accumulation of these metabolites. Notably, two metabolites (anabaenopeptin MM823 and 2-formamido-N1-(5’-phosphoribosyl) acetamidine) were consistently up-regulated, while five metabolites (chlorantene E, 3-undecylphenol, 5-heptyl-6,8-dimethyloctahydroindolizine, stearamide, and (5α)-sarmentogenin 3-*O*-[β-D-glucopyranosyl-(1→4)-α-L-rhamnopyranoside]) were consistently down-regulated across all comparisons. Moreover, nine metabolites were identified as common DAMs of the two comparisons. These findings suggest that the growth enhancement induced by *Bacillus* in delayed-transplanted strawberry plants is associated with the coordinated regulation of these metabolites, particularly those consistently altered across all comparisons.

### 3.7. KEGG Enrichment Analysis of DAMs Among the Three Comparisons

KEGG enrichment analysis was performed on the DAMs in the CK_vs_K9 comparison (Figure 6) (Supplemental Table S7). These DAMs were significantly enriched in three pathways: pantothenate and CoA biosynthesis (*p* = 0.0312), cornified envelope formation (*p* = 0.0312), and aminoacyl-tRNA biosynthesis (*p* = 0.0409). The corresponding characteristic differential metabolites were L-valine, pantetheine 4’-phosphate, pyroglutamic acid, L-glutamine, and L-asparagine (Supplemental Table S8). Among these DAMs, all except pyroglutamic acid were significantly down-regulated in K9.

KEGG enrichment analysis of DAMs in the CK_vs_KC comparison (Figure 7) revealed significant enrichment in tyrosine metabolism (*p* = 0.0470) and alpha-linolenic acid (ALA) metabolism (*p* = 0.0470) (Supplemental Table S9). The corresponding characteristic DAMs were 5-carboxymethyl-2-hydroxymuconate, hydroquinone, gentisic acid, 2,5-dihydroxybenzaldehyde, 3-amino-3-(4-hydroxyphenyl)propanoate, 13(S)-HpOTrE, 12-OPDA, 9(S)-HOTrE, 2(R)-HPOT, and 9,10-EOT (Supplemental Table S10). Among them, only 5-carboxymethyl-2-hydroxymuconate and 3-amino-3-(4-hydroxyphenyl)propanoate were down-regulated in KC, while the others were up-regulated. Notably, all ALA metabolism-related DAMs exhibited significantly higher levels in the KC group compared with the CK group.

For the CK_vs_KK comparison, DAMs were significantly enriched in the flavonoid biosynthesis pathway (*p* = 0.0104) (Figure 8) (Supplemental Table S11). The corresponding DAMs included apigenin, 5-*O*-caffeoylshikimic acid, pinocembrin, phloretin, cyanidin, chrysin, and eriodictyol (Supplemental Table S12). Among them, only 5-*O*-caffeoylshikimic acid and pinocembrin showed significantly higher levels in the KK group than in the CK group, whereas the remaining five DAMs were significantly lower in KK.

## 4. Discussion

### 4.1. Bacillus Treatments Promoted the Growth of Delayed-Transplanted Strawberry Plug Seedlings

Transplant timing is a critical determinant of post-planting survival and plant growth during strawberry production [20]. When plug seedlings remain in trays beyond the optimal window, roots coil within the confined space, resulting in a plug-bound root system [21]. Plant growth-promoting rhizobacteria (PGPR) have demonstrated considerable potential in alleviating abiotic stress and enhancing recovery in transplanted crops [38,39,40]. *Bacillus* species regulate plant development through phytohormone production and improve rhizosphere nutrient availability via organic acid, phosphatase, and siderophore secretion [41,42]. In the present study, K9, KC, and KK treatments all significantly increased plant height and fresh weight of delayed-transplanted strawberry seedlings, indicating that both strains effectively promoted the growth of delayed-transplanted strawberry. Root scanning analysis further revealed that all three treatments significantly increased root average diameter, while K9 and KC single-strain treatments also enhanced root surface area and projected area. These morphological improvements suggest that *Bacillus* inoculation promotes root architectural recovery in plug-bound seedlings, potentially restoring absorptive and metabolic functions and thereby supporting above-ground growth. These results align with previous reports that *Bacillus*-based inoculants enhance root development in strawberry and other fruit crops under stressful establishment conditions [28,30]. In addition, our cross-streaking assays revealed antagonism between the two *Bacillus* spp., which can explain why the combined treatment of KP3P9 and K13C exhibited relative weaker promoting effects compared with single-strain treatment.

### 4.2. Strain-Specific and Combined Bacillus Treatments Triggered Distinct Root Metabolic Reprogramming in Delayed-Transplanted Strawberry

Non-targeted metabolomics revealed remodeling of the root metabolic network following *Bacillus* inoculation, with differential accumulation of lipid, terpenoids, polyphenols, organic acids, nucleotides, coumarins, and sugars across treatments. The metabolic response induced by K9 was milder than that of KC and KK, which may reflect differences in their plant growth-promoting capacities. Specifically, K13C possesses additional organic phosphate-solubilizing and potassium-releasing activities not observed in KP3P9 [33]. All three *Bacillus* treatments increased root steroids contents. Phytosterols maintain membrane fluidity and integrity [43,44] and act as precursors for brassinosteroid biosynthesis [45,46]. Steroids accumulation in *Bacillus*-treated roots therefore points to improved membrane stability and brassinosteroid-dependent growth signaling, which may alleviate membrane degradation arising from root aging during delayed transplanting. Meanwhile, flavonoids, phenylpropanoids, sugars, and some other metabolites decreased across all *Bacillus* treatments. Flavonoids and phenylpropanoids are central secondary metabolites for antioxidant defense and stress adaptation [47]. Their biosynthesis is commonly activated to mitigate oxidative damage under stress [48,49]. The observed reduction of these compounds suggests a metabolic shift from defense/stress maintenance toward growth and recovery in *Bacillus*-treated delayed-transplanted strawberry roots. Moreover, the relative contents of sugars were found to be down-regulated in *Bacillus*-treated delayed-transplanted strawberry roots. The reduction in sugar content may reflect accelerated respiration and carbon consumption to sustain seedling growth [50,51], though this hypothesis awaits direct physiological validation.

Beyond these shared metabolic changes, the three treatments exhibited unique metabolic signatures. K9 and KC significantly increased polyphenols and terpenoids, which function in antioxidant protection and hormone-regulated growth processes [52,53]. KC specifically elevated alcohols, alkaloids, and coumarins. Alkaloids participate in nitrogen metabolism and plant defense [54], and coumarins mediate iron chelation and rhizosphere interactions [55], implying KC simultaneously boosts root defence and nutrient acquisition. Conversely, KK did not significantly alter polyphenols or terpenoids levels but specifically up-regulated amino acids in delayed-transplanted strawberry roots. Amino acids act as precursors for phytohormones and polyamines [56,57,58], and their accumulation upon co-inoculation may fuel secondary metabolism to support rapid cell division and shoot growth. This matches our phenotypic observation that KK produced the largest increases in plant height and crown size, even though its effects on some root traits were less pronounced than single-strain treatments. Since no antagonism or competition was detected between KP3P9 and K13C, these divergent responses may imply complex inter-strain interactions during co-inoculation, warranting further study of their molecular cross-talk.

KEGG enrichment analysis further supported strain-specific mechanisms. DAMs from CK_vs_K9 were enriched in pantothenate and CoA biosynthesis, cornified envelope formation, and aminoacyl-tRNA biosynthesis. Pantothenate participates in fatty acid metabolism and the tricarboxylic acid cycle [59,60], while aminoacyl-tRNA biosynthesis is essential for protein translation [61], indicating that K9 may facilitate root repair by stimulating protein and lipid metabolism. DAMs from CK_vs_KC were enriched in tyrosine metabolism and ALA metabolism, two pathways tightly linked to plant stress responses [62,63]. Tyrosine acts as a precursor for lignin and protective pigments [64], whereas ALA is the precursor of jasmonic acid, a key regulator of defense and development [65]. By contrast, DAMs from CK_vs_KK were specifically enriched in flavonoid metabolism. Collectively, these results demonstrate that single and combined *Bacillus* applications modulated the growth of delayed-transplanted strawberry plants through distinct, strain-specific metabolic networks rather than a common uniform mechanism.

### 4.3. Limitations

Since all seedlings received delayed transplantation without a normally transplanted control, it remains challenging to distinguish whether the observed phenotypic and metabolic changes stem from alleviation of delayed transplantation or general growth promotion by the *Bacillus* strains. Moreover, metabolomic profiling at a single time point only provides a snapshot of root metabolism, and time-series sampling is needed to characterize dynamic metabolic responses to inoculation. Although correlations were identified between *Bacillus* treatments and metabolic reprogramming, causal links between specific DAMs and growth-promoting phenotypes require targeted validation. The mechanisms responsible for divergent outcomes of single- and co-inoculation also merit further exploration at transcriptomic and phytohormonal levels. In addition, the functions of DAMs with significantly altered abundances in regulating strawberry root development and the influences of these *Bacillus* treatments on strawberry fruit quality and yield remain to be explored. Future work along these lines will advance our understanding of KP3P9 and K13C in regulating strawberry seedling performance and facilitate practical use of these *Bacillus* species.

## 5. Conclusions

This study demonstrates that KP3P9, K13C, and their combination treatments all improved the growth of delayed-transplanted strawberry (Figure 9). Non-targeted metabolomics revealed treatment-associated root metabolic reprogramming, involving shared changes in steroids, flavonoids, ketones/aldehydes/esters, lignans, nucleotides, phenylpropanoids, and sugars, alongside treatment-specific alterations in polyphenols, terpenoids, and amino acids. KEGG enrichment analysis further showed that single- and co-applications affected distinct pathways, indicating both common and strain-specific mechanisms of *Bacillus*-mediated growth promotion on delayed-transplanted strawberry. These findings offer new insight into how *Bacillus* improves the growth of delayed-transplanted strawberry.

## Figures and Tables

**Figure 1:**
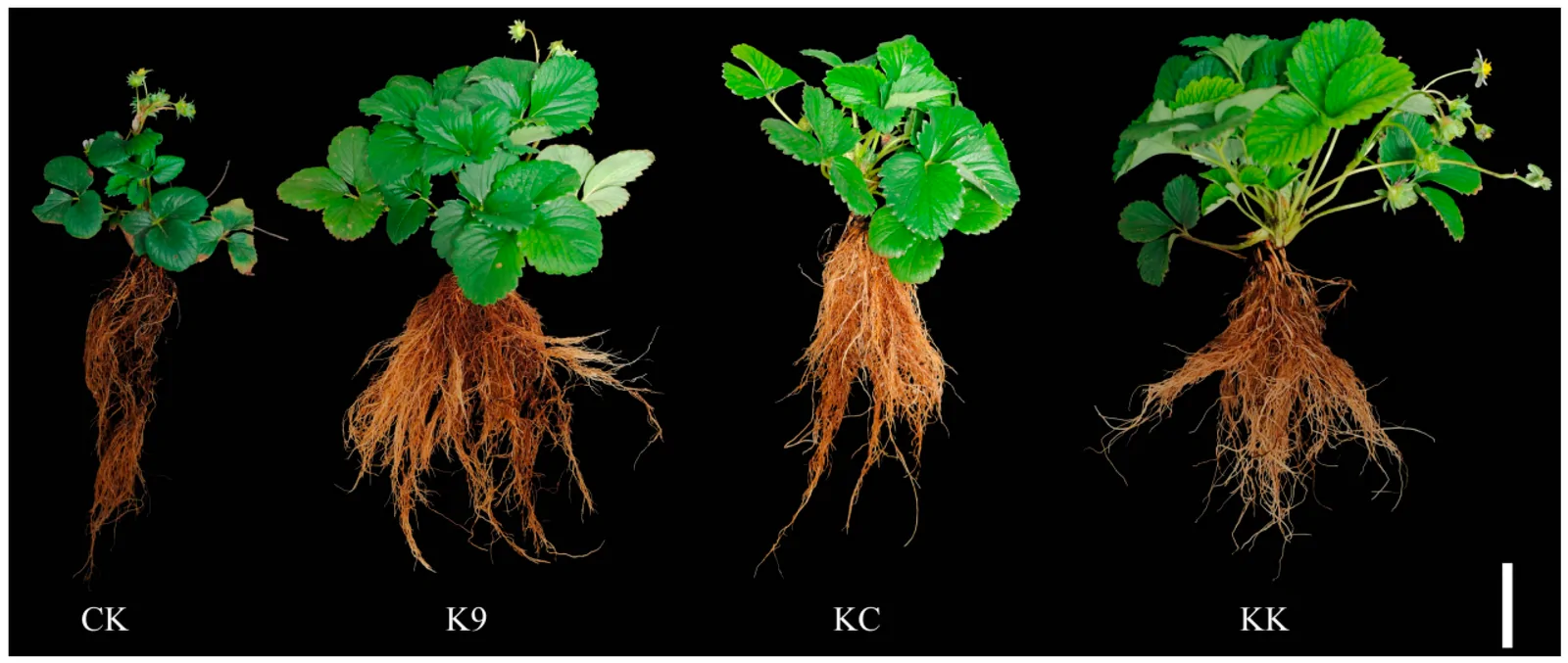
Typical phenotypes of delayed-transplanted strawberry seedlings in the four groups. Single and combined *Bacillus* strains were used to treat 12-week-old ‘Benihoppe’ strawberry (*Fragaria* × *ananassa* Duch.) plug seedlings (had been delayed-transplanted for approximately 5–8 weeks), and plant phenotypes were observed at three months post-transplantation. CK: non-treated control; K9: KP3P9 treatment; KC: K13C treatment; KK: combined KP3P9 and K13C treatment. Scale bar = 5 cm.

**Figure 2:**
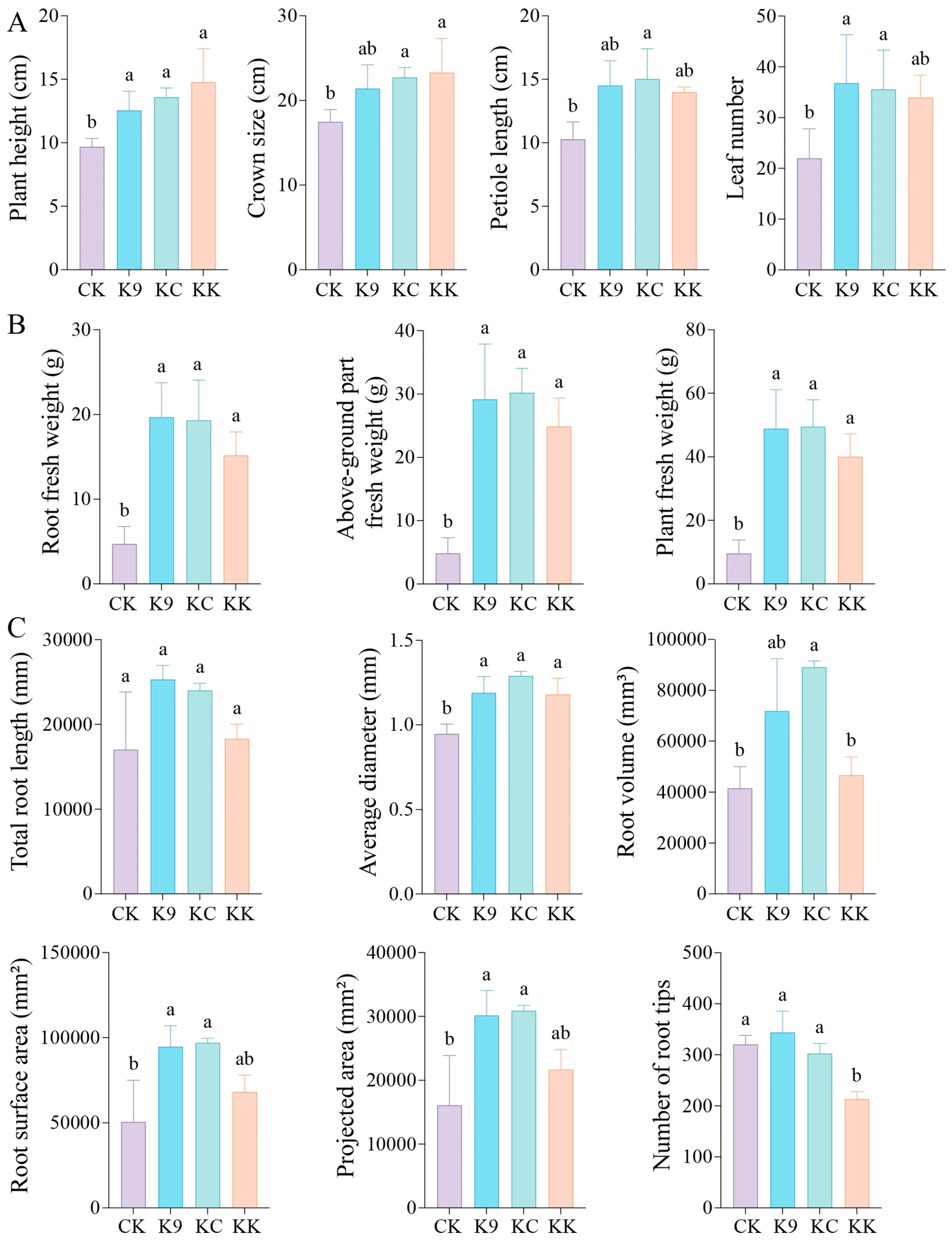
Effects of K9, KC, and KK treatments on the growth- (**A**), fresh weight- (**B**), and root architecture-related (**C**) parameters of delayed-transplanted strawberry seedlings at three months post inoculation. CK: non-treated control; K9: KP3P9 treatment; KC: K13C treatment; KK: combined KP3P9 and K13C treatment. Values are expressed as means ± standard deviations (*n* = 24 in (**A**), *n* = 6 in (**B**,**C**)). Different lowercase letters above the columns denote significant differences among groups (*p* < 0.05, one-way ANOVA followed by Duncan’s post hoc test).

**Figure 3:**
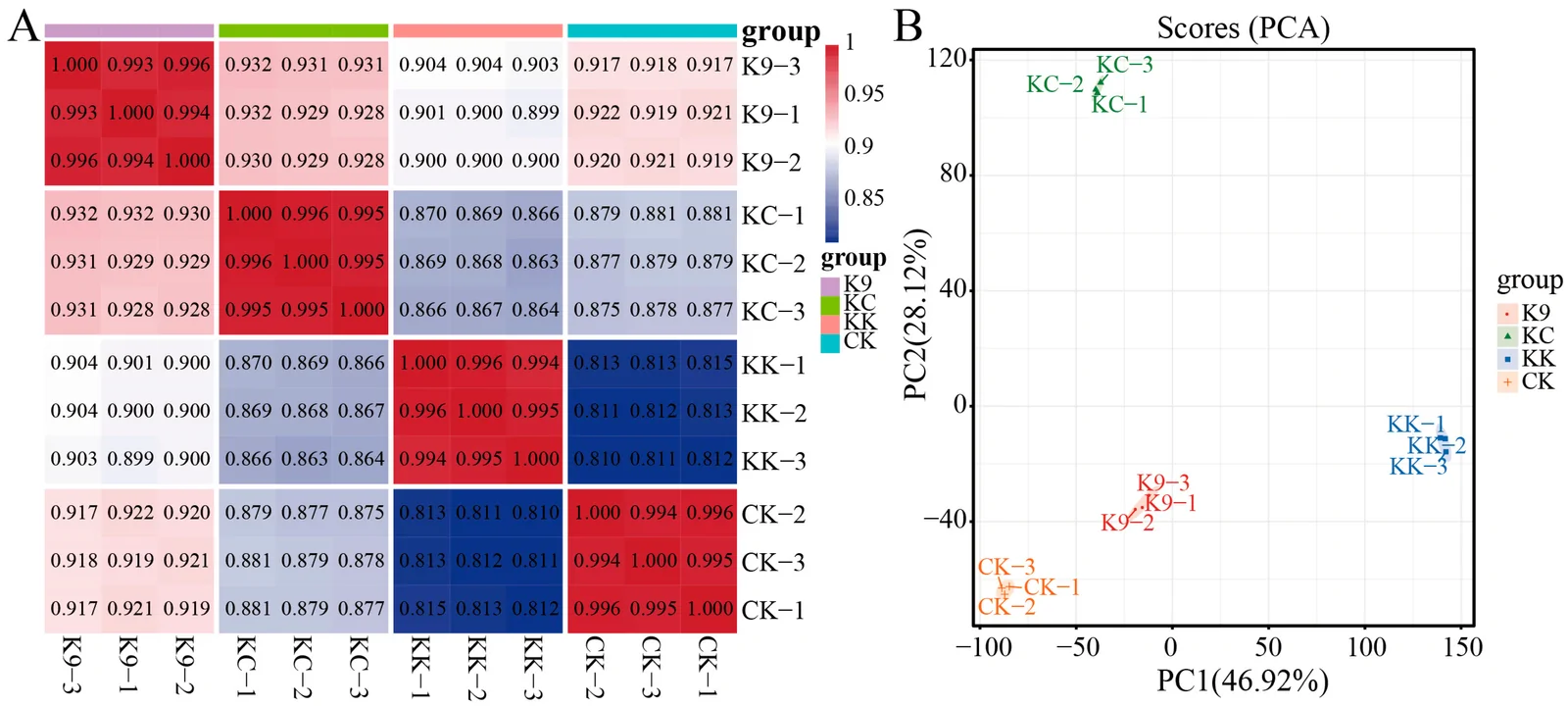
Correlation (**A**) and PCA (**B**) analysis of the strawberry root metabolome across the four treatment groups. CK: non-treated control; K9: KP3P9 treatment; KC: K13C treatment; KK: combined KP3P9 and K13C treatment. For each group, three biological replicates were made.

**Figure 4:**
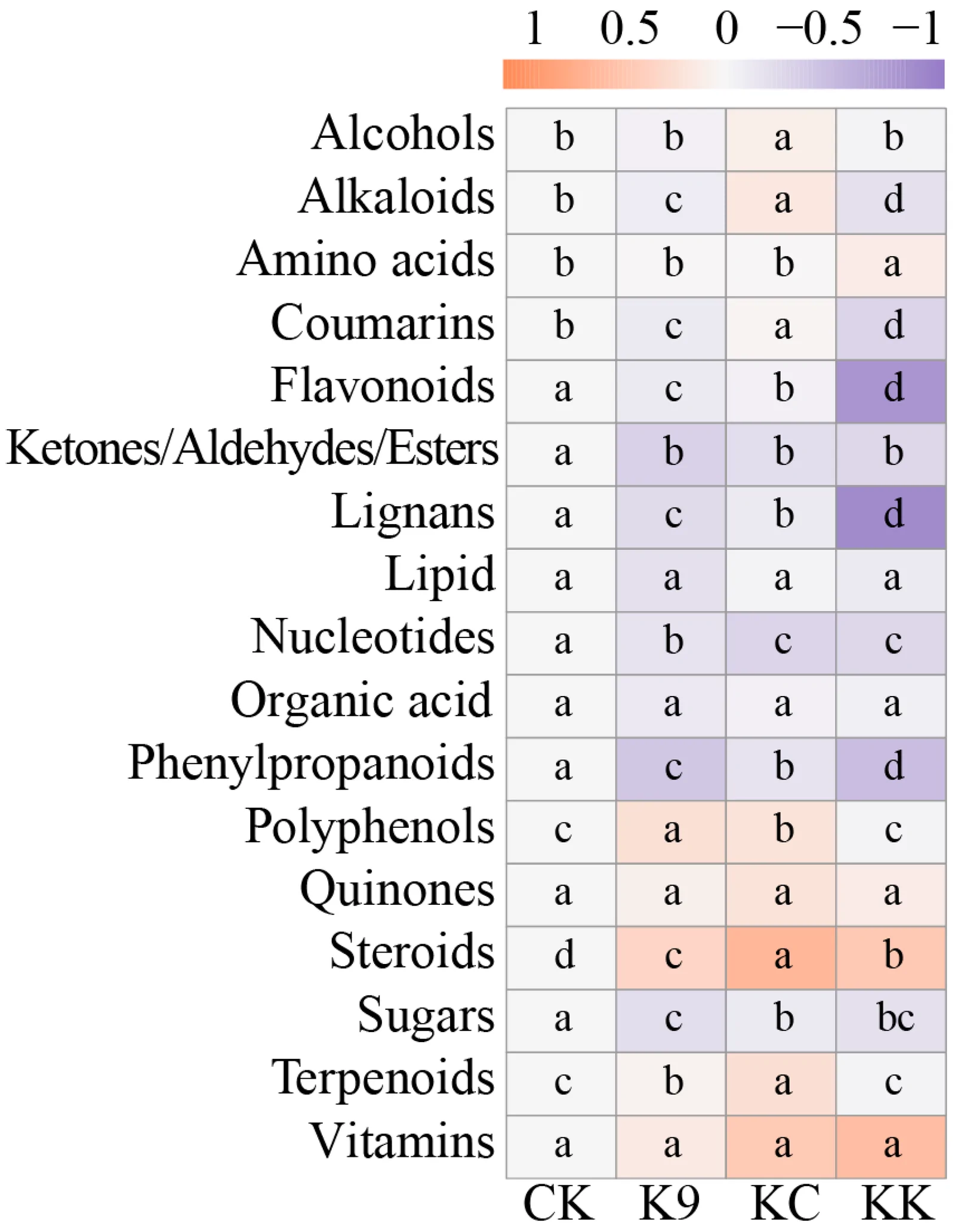
Heatmap analysis showing the relative contents of Class I-level metabolites in the roots of strawberry plants from the four groups. CK: non-treated control; K9: KP3P9 treatment; KC: K13C treatment; KK: combined KP3P9 and K13C treatment. The Log_2_(fold change) values calculated from average total peak areas of Class I-level metabolites for the K9, KC, and KK groups relative to CK were employed for heatmap generation (*n* = 3). Different lowercase letters in the same row denote significant differences among groups (*p* < 0.05, one-way ANOVA followed by Duncan’s post hoc test).

**Figure 5:**
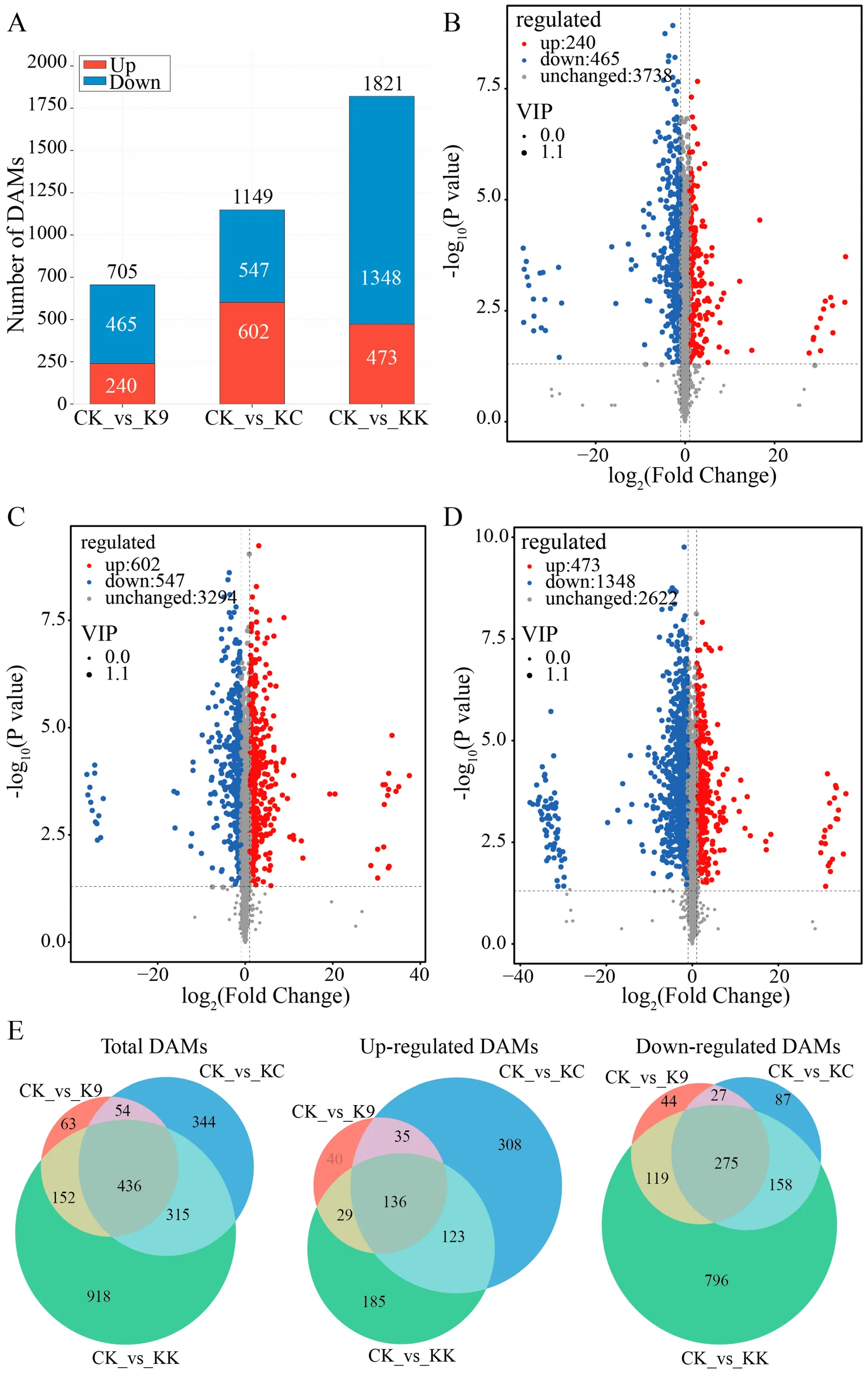
Identification (**A**–**D**) and Venn analysis (**E**) of differentially accumulated metabolites (DAMs) across the three comparisons. (**A**): Bar chart for the numbers of DAMs identified in the CK_vs_K9, CK_vs_KC, and CK_vs_KK comparisons; (**B**–**D**): Volcano plots of DAMs identified in the CK_vs_K9, CK_vs_KC, and CK_vs_K9 comparison, respectively; (**E**): Venn diagram of all DAMs, up-regulated DAMs, and down-regulated DAMs among CK_vs_K9, CK_vs_KC, and CK_vs_KK comparisons.

**Figure 6:**
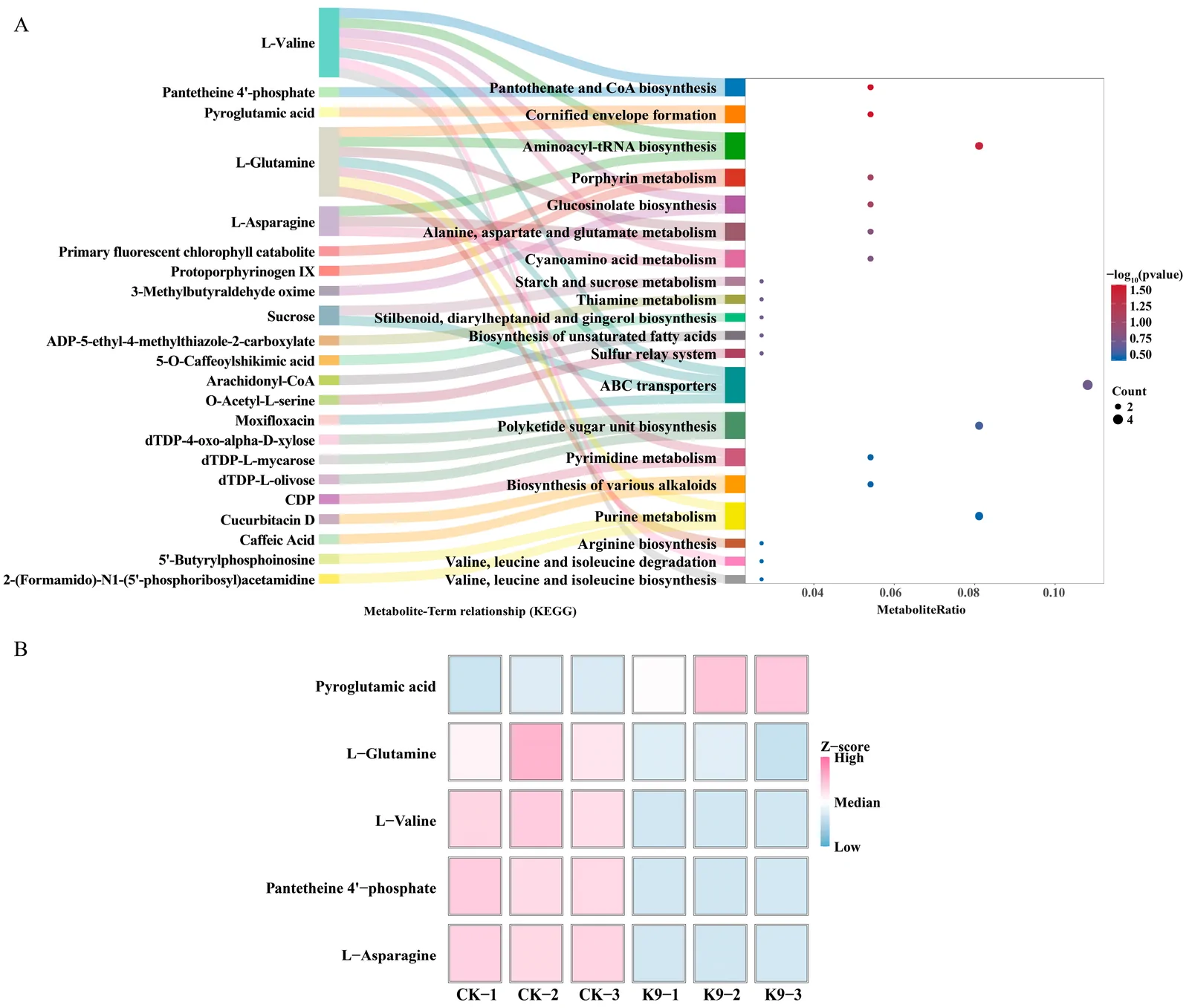
KEGG enrichment analysis of DAMs in the CK_vs_K9 comparison. In (**A**), the Sankey diagram illustrates the correspondence between DAMs and KEGG pathways, while the bubble plot represents the pathways enriched by DAMs. Bubble size represents the number of DAMs, and the color gradient represents −log_10_(*p*-value), with darker red indicating higher enrichment significance. In (**B**), the heatmap shows a comparison of DAM contents involved in significantly enriched KEGG pathways between the CK and K9 groups.

**Figure 7:**
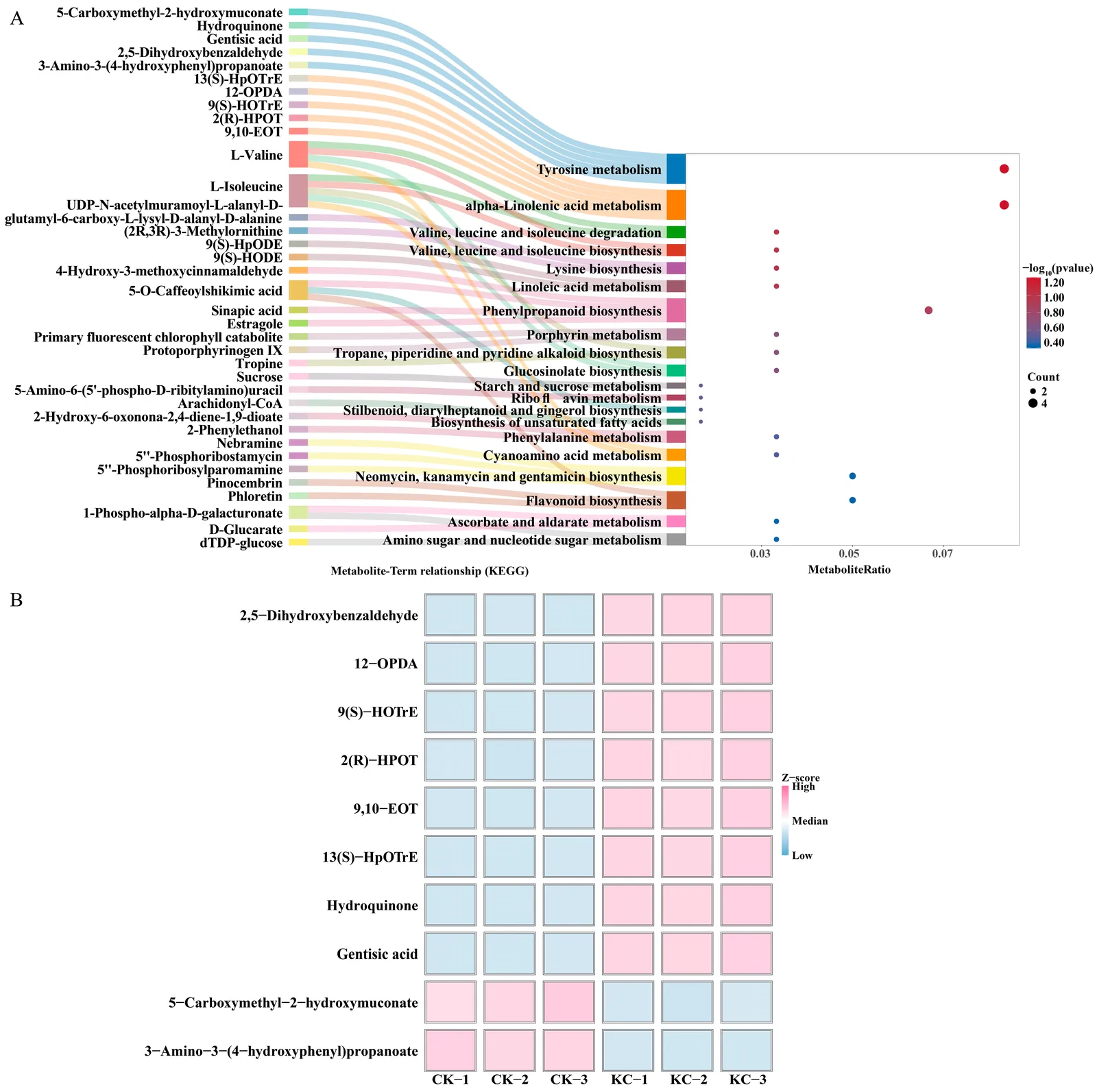
KEGG enrichment analysis of DAMs in the CK_vs_KC comparison. In (**A**), the Sankey diagram illustrates the correspondence between DAMs and KEGG pathways, while the bubble plot represents the pathways enriched by DAMs. Bubble size represents the number of DAMs, and the color gradient represents −log_10_(*p*-value), with darker red indicating higher enrichment significance. In (**B**), the heatmap shows a comparison of DAM contents involved in significantly enriched KEGG pathways between the CK and KC groups.

**Figure 8:**
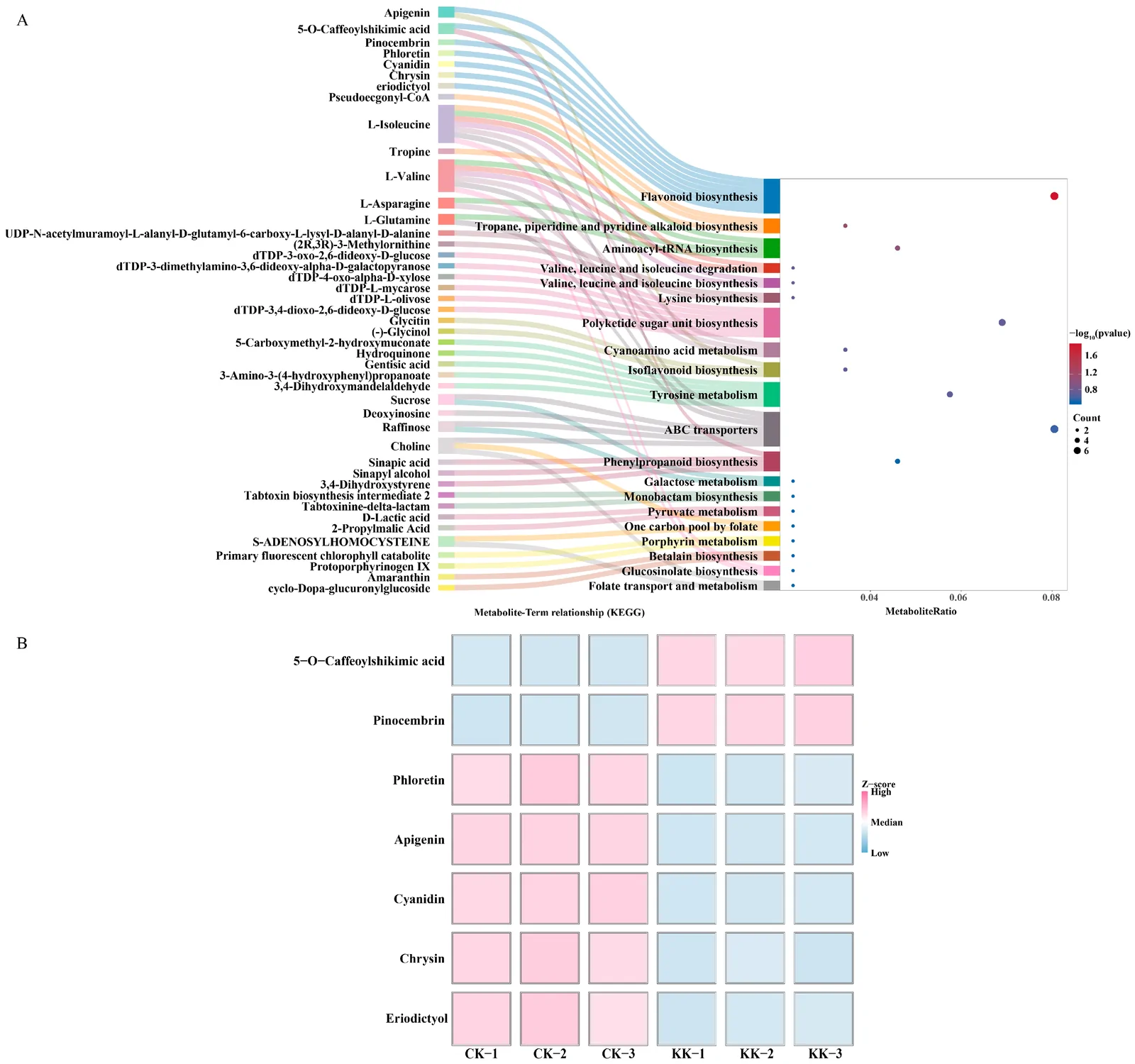
KEGG enrichment analysis of DAMs in the CK_vs_KK comparison. In (**A**), the Sankey diagram illustrates the correspondence between DAMs and KEGG pathways, while the bubble plot represents the pathways enriched by DAMs. Bubble size represents the number of DAMs, and the color gradient represents −log_10_(*p*-value), with darker red indicating higher enrichment significance. In (**B**), the heatmap shows a comparison of DAM contents involved in significantly enriched KEGG pathways between the CK and KK groups.

**Figure 9:**
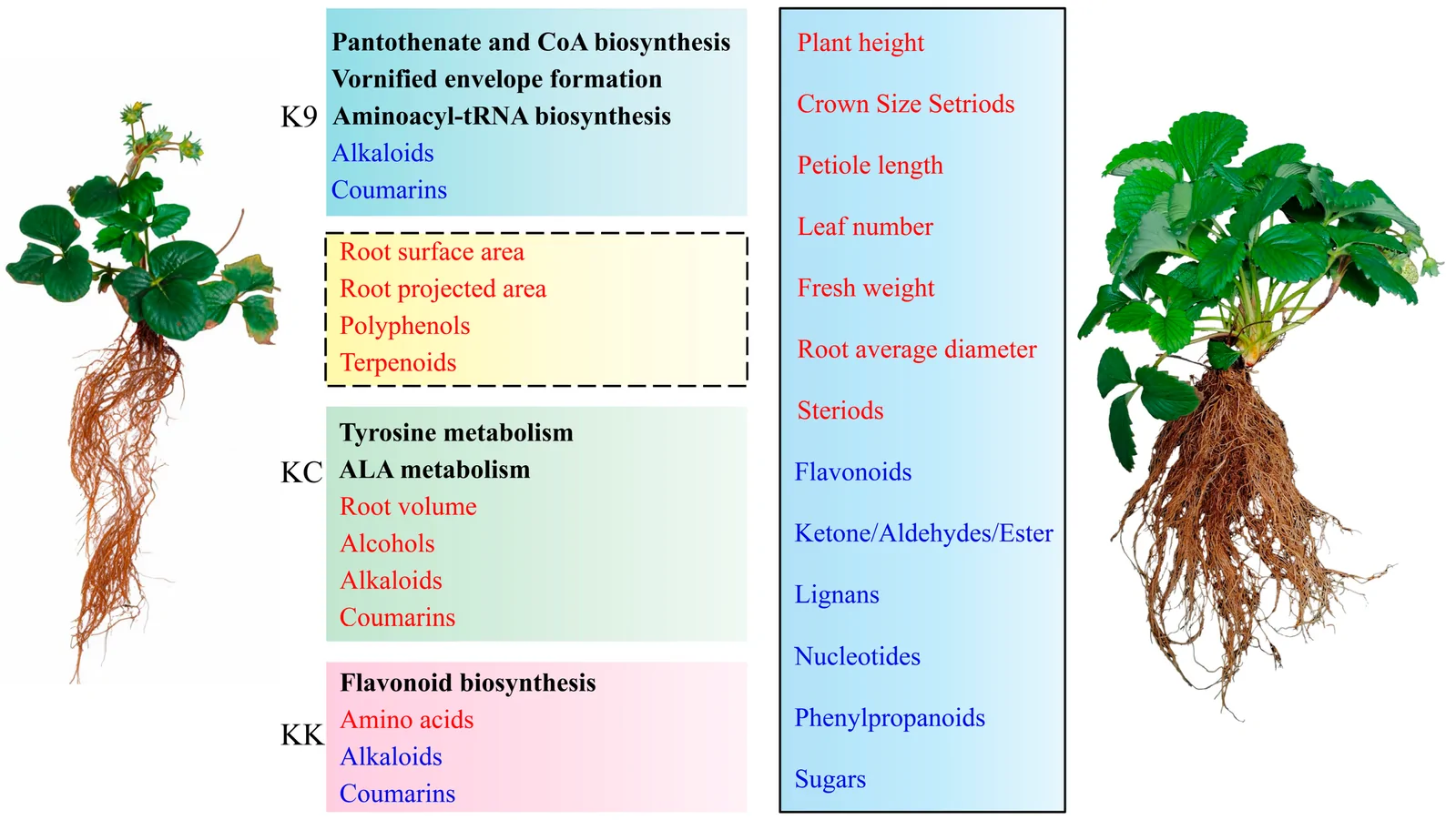
Schematic overview of distinct root metabolic reprogramming underlying *Bacillus*-mediated growth enhancement in delayed-transplanted strawberry seedlings. Solid-line boxes indicate pathways and metabolites commonly altered across all three treatment groups; dashed-line boxes indicate those commonly altered in both K9 and KC. Bold text denotes KEGG pathways significantly enriched for DAMs in each group. Red and blue text indicates up- and down-regulation, respectively, compared with the CK group. ALA: Alpha-linolenic acid.

**Table 1:** Top 20 up- and down-regulated DAMs in the CK_vs_K9, CK_vs_KC, and CK_vs_KK comparisons. Bold metabolites are shared across all three comparisons, and underlined metabolites are shared between two comparisons. FC: Fold change. VIP: Variable importance in projection.

Comparison	ID	Metabolite Name	Class I	Class II	log2FC	*p*-Value	VIP
CK_vs_K9	neg_7199	**Anabaenopeptin MM823**	Organic Acid	Carboxylic Acids	35.88	1.92 × 10^−4^	1.16
	neg_4081	5,6-dehydro Arachidonic Acid	--	--	35.70	2.03 × 10^−3^	1.16
	pos_1294	Ruine	Polyphenols	Phenolic Glycosides	33.05	9.92 × 10^−3^	1.14
	neg_7400	2alpha,12-dihydroxy-(1alphaH)-1,2-dihydro-senecionane-11,16-dione	Ketones/Aldehydes/Esters	Polyketide	32.95	2.43 × 10^−3^	1.16
	neg_8271	24-methylene-24(25)-dihydrolanosten-3-one	Ketones/Aldehydes/Esters	Other	32.55	1.58 × 10^−3^	1.16
	pos_6200	**2-(Formamido)-N1-(5′-phosphoribosyl)acetamidine**	Others	Other	31.34	1.92 × 10^−3^	1.16
	pos_5987	3′-Fluoro-3′-deoxythymidine Monophosphate	Nucleotides	Pyrimidine Nucleotide	30.95	2.90 × 10^−3^	1.16
	neg_8434	Ethyl Ricinoleate	Lipid	Fatty Acid Esters	30.28	2.51 × 10^−2^	1.11
	pos_4776	17-(4-Amino-3-methylbutyl)-9,27-dihydroxy-6,10,13,27-tetramethyl-22-methylidene-14-(4-methyl-5-oxooxolan-2-yl)-28,29,30-trioxapentacyclo[22.3.1.11,4.14,7.012,17]triaconta-10,13-dien-18-one	Ketones/Aldehydes/Esters	Ketal	30.28	4.68 × 10^−3^	1.15
	neg_6481	methyl 3-hydroxypentadecanoate	Lipid	Fatty Alcohol	29.44	7.56 × 10^−3^	1.15
	neg_5885	25-Cinnamoyl-Vulgaroside	Lipid	Isoprene lipid	−32.45	4.49 × 10^−4^	1.16
	pos_5250	3-Epicabraleahydroxylactone	Terpenoids	Triterpenoids	−33.79	4.18 × 10^−3^	1.15
	pos_936	Man2GlcNAcFucGlcNAc	Sugars	Oligosaccharides	−33.86	8.95 × 10^−3^	1.14
	pos_4913	Psoralidin oxide	--	--	−33.91	1.73 × 10^−3^	1.16
	neg_6355	**Chlorantene E**	Lipid	Isoprene lipid	−34.96	8.51 × 10^−4^	1.16
	pos_4469	**Phenol, 3-undecyl-**	Polyphenols	Other	−35.31	5.48 × 10^−4^	1.16
	pos_5487	**5-heptyl-6,8-dimethyloctahydroindolizine**	--	--	−35.51	2.45 × 10^−4^	1.16
	pos_5728	**Stearamide**	--	--	−35.98	3.69 × 10^−4^	1.16
	neg_7934	12-O-methylvolkensin	Terpenoids	Triterpenoids	−36.10	5.78 × 10^−3^	1.15
	neg_8136	**(5alpha)-sarmentogenin 3-O-[beta-D-glucopyranosyl-(1->4)-alpha-L-rhamnopyranoside]**	Lipid	Steroid	−36.20	1.23 × 10^−4^	1.16
CK_vs_KC	neg_7199	**Anabaenopeptin MM823**	Organic Acid	Carboxylic Acids	37.51	1.31 × 10^−4^	1.12
	neg_8271	24-methylene-24(25)-dihydrolanosten-3-one	Ketones/Aldehydes/Esters	Other	35.09	2.37 × 10^−4^	1.12
	neg_7484	4H-Furo[2,3-b]pyran-4-one,2,3-dihydro-6-(4-hydroxyphenyl)-2,3,3-trimethyl-, (-)-	Others	Furans	34.46	3.05 × 10^−4^	1.12
	neg_6481	Methyl 3-hydroxypentadecanoate	Lipid	Fatty Alcohol	33.54	1.52 × 10^−5^	1.12
	pos_5835	2H-Furo[3,2-b]naphtho[2,3-d]pyran-2,6,11-trione,3,3a,5,11b-tetrahydro-7-hydroxy-5-methyl-8-[2,3,6-trideoxy-3-(dimethylamino)-b-D-arabino-hexopyranosyl]-,(3aR,5R,11bR)-	--	--	32.88	1.71 × 10^−2^	1.08
	neg_8434	Ethyl Ricinoleate	Lipid	Fatty Acid Esters	32.79	1.16 × 10^−4^	1.12
	neg_6571	11-hydroxyhexadecanoic acid	Lipid	Fatty Acids	32.76	2.75 × 10^−4^	1.12
	pos_8150	2(5H)-Furanone, 3-[(11S)-11-hydroxy-11-[(2S,5R)-tetrahydro-5-[(1R)-1-hydroxypentadecyl]-2-furanyl]undecyl]-5-methyl-, (5S)-	Lipid	Fatty Acyls	32.69	1.85 × 10^−2^	1.08
	pos_4776	17-(4-Amino-3-methylbutyl)-9,27-dihydroxy-6,10,13,27-tetramethyl-22-methylidene-14-(4-methyl-5-oxooxolan-2-yl)-28,29,30-trioxapentacyclo[22.3.1.11,4.14,7.012,17]triaconta-10,13-dien-18-one	Ketones/Aldehydes/Esters	Ketal	32.55	3.80 × 10^−4^	1.12
	pos_6200	**2-(Formamido)-N1-(5′-phosphoribosyl)acetamidine**	Others	Other	32.01	2.13 × 10^−4^	1.12
	pos_5250	3-Epicabraleahydroxylactone	Terpenoids	Triterpenoids	−33.79	4.18 × 10^−3^	1.11
	pos_4913	Psoralidin oxide	--	--	−33.91	1.73 × 10^−3^	1.11
	pos_703	CIDP-RIBOSE	Nucleotides	Purine Nucleosides	−34.24	1.57 × 10^−3^	1.11
	pos_5544	1,3,6,9-Nonadecatetraene,(3Z,6Z,9Z)-	Others	Hydrocarbons	−34.40	7.44 × 10^−5^	1.12
	neg_5408	Avermectin B1b aglycone	Others	Other	−34.41	1.15 × 10^−4^	1.12
	neg_6355	**Chlorantene E**	Lipid	Isoprene lipid	−34.96	8.51 × 10^−4^	1.12
	pos_4469	**Phenol, 3-undecyl-**	Polyphenols	Other	−35.31	5.48 × 10^−4^	1.12
	pos_5487	**5-heptyl-6,8-dimethyloctahydroindolizine**	--	--	−35.51	2.45 × 10^−4^	1.12
	pos_5728	**Stearamide**	--	--	−35.98	3.69 × 10^−4^	1.12
	neg_8136	**(5alpha)-sarmentogenin 3-O-[beta-D-glucopyranosyl-(1->4)-alpha-L-rhamnopyranoside]**	Lipid	Steroid	−36.20	1.23 × 10^−4^	1.12
CK_vs_KK	neg_7484	4H-Furo[2,3-b]pyran-4-one,2,3-dihydro-6-(4-hydroxyphenyl)-2,3,3-trimethyl-, (-)-	Others	Furans	35.71	2.02 × 10^−4^	1.08
	neg_7199	**Anabaenopeptin MM823**	Organic Acid	Carboxylic Acids	35.12	6.15 × 10^−3^	1.07
	pos_6200	**2-(Formamido)-N1-(5′-phosphoribosyl)acetamidine**	Others	Other	33.98	5.10 × 10^−4^	1.08
	pos_4997	2-Propenal, 3-(1,3-benzodioxol-5-yl)-	--	--	33.78	8.54 × 10^−4^	1.08
	pos_5987	3′-Fluoro-3′-deoxythymidine Monophosphate	Nucleotides	Pyrimidine Nucleotide	33.49	1.45 × 10^−4^	1.08
	pos_5991	1(2H)-Isoquinolinone,3,4-dihydro-6,7-dihydroxy-	Ketones/Aldehydes/Esters	Other	33.45	8.33 × 10^−4^	1.08
	neg_8167	(Z)-Icos-9-en-1-ol	Lipid	Fatty Acyls	33.30	1.27 × 10^−4^	1.08
	pos_1762	(2S,3R)-(-)-3-hydroxybaikiain	Organic Acid	Carboxylic Acids	33.05	2.57 × 10^−4^	1.08
	pos_2757	FRAXETIN	Coumarins	Other	32.58	8.25 × 10^−3^	1.06
	neg_4081	5,6-dehydro Arachidonic Acid	--	--	32.10	1.66 × 10^−2^	1.05
	pos_930	2-(3,4-dihydroxyphenyl)ethyl O-beta-D-glucopyranosyl-(1->3)-O-[beta-D-glucopyranosyl-(1->6)]-4-O-(E)-feruloyl-beta-D-glucopyranoside	Sugars	Oligosaccharides	−34.89	8.79 × 10^−4^	1.08
	neg_6355	**Chlorantene E**	Lipid	Isoprene lipid	−34.96	8.51 × 10^−4^	1.08
	neg_6728	15-O-acetyl-ent-15beta,16beta,17,19-tetrahydroxykaurane 17-O-beta-D-glucopyranoside	Terpenoids	Terpene Glycoside	−35.02	6.34 × 10^−4^	1.08
	pos_4469	**Phenol, 3-undecyl-**	Polyphenols	Other	−35.31	5.48 × 10^−4^	1.08
	pos_5487	**5-heptyl-6,8-dimethyloctahydroindolizine**	--	--	−35.51	2.45 × 10^−4^	1.08
	neg_1090	Arachidonyl-CoA	Nucleotides	Glycosides	−35.79	2.95 × 10^−4^	1.08
	pos_5728	**Stearamide**	--	--	−35.98	3.69 × 10^−4^	1.08
	neg_8136	**(5alpha)-sarmentogenin 3-O-[beta-D-glucopyranosyl-(1->4)-alpha-L-rhamnopyranoside]**	Lipid	Steroid	−36.20	1.23 × 10^−4^	1.08
	neg_1134	Hydracyanoside D	Terpenoids	Terpene Glycoside	−37.09	3.68 × 10^−4^	1.08
	pos_4364	Methylnissolin	Polyphenols	Other	−37.75	3.33 × 10^−4^	1.08

## Data Availability

The original contributions presented in this study are included in the article/Supplementary Material. Further inquiries can be directed to the corresponding author.

## Supplementary Materials

The following supporting information can be downloaded at: https://www.mdpi.com/article/10.3390/microorganisms14102135/s1, Supplemental Figure S1: Cross-streaking assay results of the antagonism between KP3P9 and K13C. (A): the antagonistic activity of KP3P9 against K13C; (B) the antagonistic activity of K13C against KP3P9. Scale bar = 2 cm. Supplemental Table S1: Information for the metabolites identified in strawberry roots. Supplemental Table S2: The total peak areas of the Class I-level metabolites in roots of delayed-transplanted strawberry. Supplemental Table S3: The 705 DAMs identified in the CK_vs_K9 comparison. Supplemental Table S4: The 1149 DAMs identified in the CK_vs_KC comparison. Supplemental Table S5: The 1821 DAMs identified in the CK_vs_KK comparison. Supplemental Table S6: Common DAMs identified among the three comparisons. Supplemental Table S7: KEGG enrichment analysis results of DAMs identified in the CK_vs_K9 comparison. Supplemental Table S8: DAMs involved in the significantly enriched KEGG pathways from the CK_vs_K9 comparison. Supplemental Table S9: KEGG enrichment analysis results of DAMs identified in the CK_vs_KC comparison. Supplemental Table S10: DAMs involved in the significantly enriched KEGG pathways from the CK_vs_KC comparison. Supplemental Table S11: KEGG enrichment analysis results of DAMs identified in the CK_vs_KK comparison. Supplemental Table S12: DAMs involved in the significantly enriched KEGG pathways from the CK_vs_KK comparison.

## Abbreviations

The following abbreviations are used in this manuscript:

ALA	Alpha-linolenic acid
CFU	Colony-forming units
CK	Non-treated control
DAM	Differentially accumulated metabolites
EC	Electrical conductivity
FC	Fold change
Hpi	Hour post inoculation
K9	KP3P9 treatment
KC	K13C treatment
KEGG	Kyoto Encyclopedia of Genes and Genomes
KK	KP3P9 and K13C treatment
LB	Luria–Bertani
MS	Mass spectrometer
OD	Optical density
PCA	Principal component analysis
PGPB	Plant growth-promoting bacteria
PGPM	Plant growth-promoting microorganism
PGPR	Plant growth-promoting rhizobacteria
Rpm	Revolutions per minute
SD	Standard deviation
UPLC	Ultra-performance liquid chromatography
VIP	Variable importance in projection
